# Receipt of needed care coordination and emergency department utilization among US children with chronic physical pain: a pooled analysis of the 2022–2024 National Survey of Children's Health

**DOI:** 10.3389/fpain.2026.1994046

**Published:** 2026-10-07

**Authors:** Tongming Cheng, Ziming Cai, Xiaonan Zhou, Ciai Lai, Lizhen Huang, Wei He

**Affiliations:** 1 Pediatrics, Shenzhen Hospital (Futian) of Guangzhou University of Chinese Medicine, Shenzhen, Guangdong, China; 2 State Key Laboratory of Traditional Chinese Medicine Syndrome, The Second Clinical Medical College of Guangzhou University of Chinese Medicine, Guangzhou, Guangdong, China; 3 School of Public Health and Management, Guangzhou University of Chinese Medicine, Guangzhou, Guangdong, China

**Keywords:** care coordination, child health, chronic pain, emergency department utilization, healthcare access, National Survey of Children's Health (NSCH), pediatric health services

## Abstract

**Background:**

Children with chronic physical pain often require complex, multidisciplinary services across medical, behavioral, and community systems. However, whether receipt of needed care coordination is associated with acute healthcare utilization and care access in this population remains poorly characterized.

**Methods:**

We conducted a pooled cross-sectional analysis of the 2022–2024 National Survey of Children's Health (NSCH). The analytic cohort included US children aged 6–17 years with caregiver-reported chronic physical pain who had an identified need for care coordination. The exposure was receipt of needed care coordination. The primary outcome was all-cause emergency department (ED) utilization (≥1 visit) in the prior 12 months. Survey-weighted multivariable logistic regression was used, with a minimally adjusted model (sociodemographic factors) and a fully adjusted model (additionally including insurance status, anxiety, depression, and functional difficulties) reported side by side.

**Results:**

Among 6,326 eligible children (representing an average of 878,491 children annually), weighted 12-month ED visit rates were 29.0% (95% CI, 25.4%–32.5%) for those who received needed care coordination versus 40.0% (95% CI, 36.4%–43.7%) for those who did not. In the minimally adjusted model, receipt of needed care coordination was associated with lower odds of ED utilization (adjusted OR [aOR] 0.61; 95% CI, 0.48–0.76; *P* < .001; *n* = 6,310). This association persisted in the fully adjusted model (aOR 0.69; 95% CI, 0.54–0.87; *P* = .0019; *n* = 6,145) and was consistent for recurrent ED visits (≥2 visits: aOR 0.54; 95% CI, 0.36–0.79; *P* = .0015). Receipt of care coordination was also associated with substantially lower odds of unmet healthcare needs (aOR 0.32; 95% CI, 0.23–0.45) and forgone mental health care (aOR 0.21; 95% CI, 0.13–0.33). The estimate for hospital stay was imprecise and included the null (aOR 0.61; 95% CI, 0.37–1.01; *P* = .053).

**Conclusion:**

Among US children with chronic physical pain who needed care coordination, receipt of needed care coordination was associated with lower odds of all-cause ED use and reported healthcare access gaps. These cross-sectional findings do not establish temporal ordering or causal effects and should be evaluated in prospective studies.

## 1 Introduction

Pediatric chronic physical pain represents a pervasive and debilitating public health challenge, precipitating profound functional impairment, school absenteeism, and psychological comorbidity (1). Navigating pediatric chronic pain requires continuous orchestration across primary care, subspecialty medicine, physical therapy, and behavioral health services (2). However, modern pediatric healthcare systems remain markedly fragmented. When multi-system care fails to communicate, ambulatory management frequently becomes fragmented, and families may rely more heavily on hospital emergency departments (EDs) for acute crisis stabilization (3). While ED encounters provide transient symptom relief, they are fundamentally ill-equipped for longitudinal, rehabilitative pain care, exposing children to episodic and uncoordinated interventions while imposing substantial avoidable costs on families and health systems (4, 5).

Structured care coordination has emerged as an essential health-system mechanism designed to integrate disparate medical, psychological, and social resources into a continuous, patient-centered care pathway (6). By proactively synchronizing inter-provider communication, facilitating timely subspecialty referrals, and establishing stable ambulatory treatment plans, effective coordination may help address symptom escalation before acute medical crises develop (7, 8). Nevertheless, prior investigations have predominantly focused on broad, heterogeneous cohorts of children with special healthcare needs or remained confined to localized single-center programs (9, 10). A decisive empirical gap persists regarding whether successfully fulfilling caregiver-identified care coordination needs translates into tangible reductions in acute healthcare reliance at the national population scale among youth living with chronic physical pain.

To address this knowledge gap, we examined a contemporary, nationally representative pooled cohort from the 2022–2024 National Survey of Children's Health (NSCH), comprising 6,326 children aged 6–17 years with caregiver-reported chronic physical pain and identified care coordination needs (11). Rather than evaluating broad populations without clear healthcare navigation requirements, focusing specifically on children with documented coordination needs establishes a rigorous benchmark to evaluate the real-world value of successfully delivered care (12). Using survey-weighted multivariable logistic regression, we evaluated 12-month all-cause ED utilization and recurrent ED encounters, while systematically accounting for sociodemographic disparities, insurance adequacy, functional limitations, and co-occurring anxiety and depression (13).

In this pooled national sample, receipt of needed care coordination was associated with lower odds of all-cause ED use, recurrent ED use, and reported healthcare access gaps. These associations are interpreted as descriptive evidence of differences in care experiences, not as proof that care coordination caused lower acute-care use.

## 2 Methods

### 2.1 Study design and data source

This pooled cross-sectional investigation utilized independent annual public-use topical data files from the 2022, 2023, and 2024 National Survey of Children's Health (NSCH) (14, 15). The NSCH is a nationally representative survey administered by the US Census Bureau on behalf of the Health Resources and Services Administration's Maternal and Child Health Bureau. The survey collects comprehensive caregiver-reported data regarding physical and emotional health, healthcare encounters, and social determinants among non-institutionalized US children aged 0 to 17 years. To avoid duplicate sampling units and correlation across overlapping multi-year rolling samples, each single-year file was obtained and pooled independently.

To account for multi-year pooling and produce valid annual-average population estimates, the NSCH selected-child sampling weight was divided by three. Complex survey design features, including strata and household primary sampling clusters, were explicitly nested within survey years to reflect the independent probability sampling design executed each cycle. Because NSCH data files are fully anonymized and publicly accessible, this study did not constitute human subjects research requiring institutional review board review.

### 2.2 Study population and cohort selection

The target population comprised non-institutionalized US children aged 6 to 17 years with caregiver-reported chronic physical pain and an identified need for care coordination. From an initial pooled sample of 160,640 respondents across the 2022–2024 surveys, restriction to school-aged children and adolescents aged 6 to 17 years yielded 105,534 individuals. Children with missing or indeterminate chronic pain responses were removed (*n* = 580), leaving 104,954 children with complete chronic physical pain assessments.

Within this denominator, 8,342 children were identified as having caregiver-reported chronic physical pain, while 96,612 children were excluded because their caregivers did not report chronic physical pain. The characteristics of these two groups are compared in Supplementary Table S5. The final analytic cohort was defined by restricting this group to children whose caregivers reported a need for healthcare coordination across providers and supplied a valid response regarding whether that coordination was received. Children with unperceived coordination needs or missing coordination status were excluded, establishing an unweighted analytic cohort of 6,326 children. The complete stepwise participant selection process is illustrated in Figure 1.

**Figure 1:**
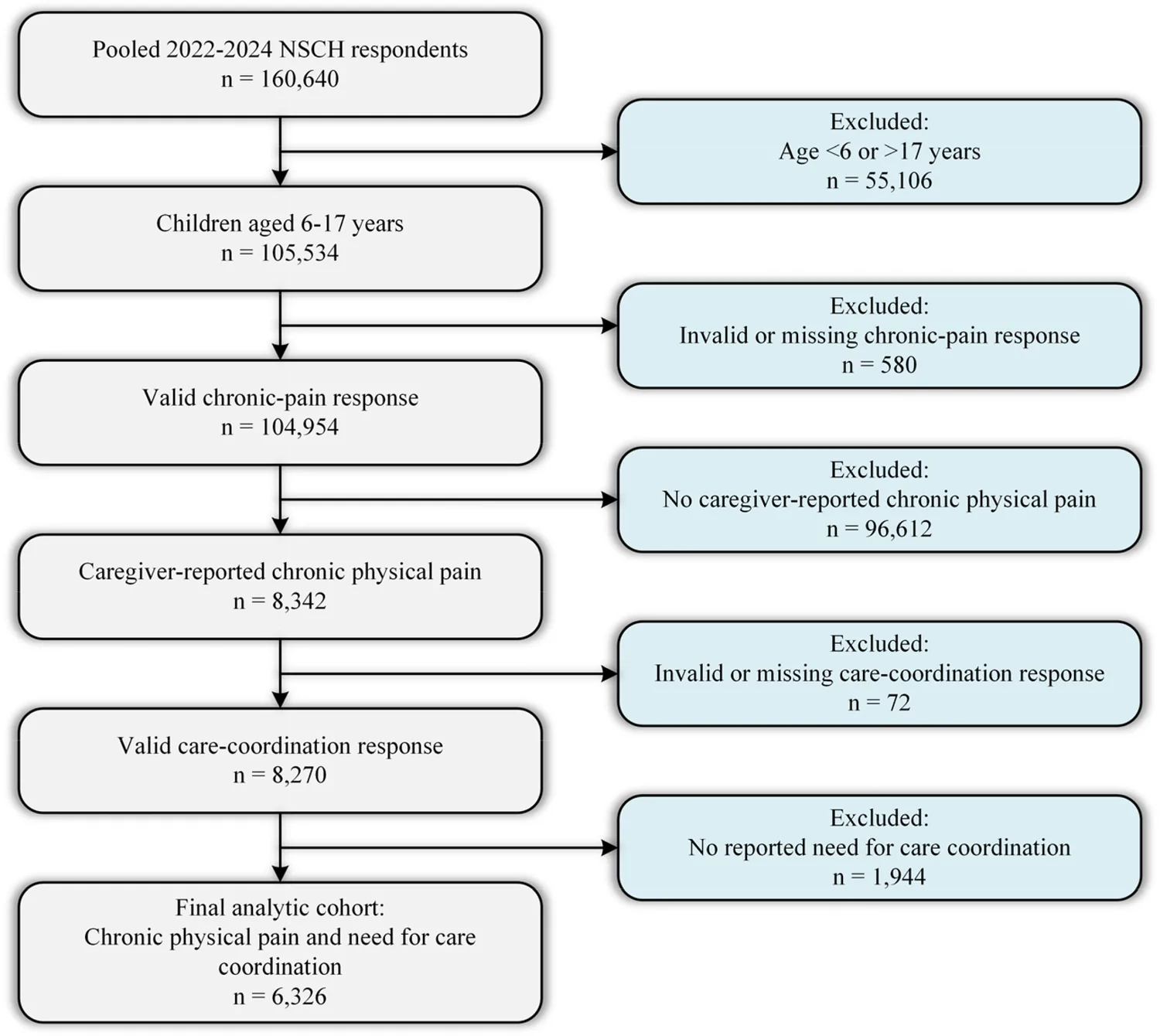
Study population selection flowchart.

### 2.3 Measures and definitions

The primary exposure was receipt of needed care coordination (16). Caregivers who affirmed that their child needed help arranging or coordinating care among two or more doctors, specialists, or service providers were categorized into two comparative groups: those who received all needed care coordination versus those who experienced unmet coordination needs.

The primary study outcome was any all-cause emergency department (ED) visit within the preceding 12 months, operationalized as a binary indicator comparing one or more ED visits against none. Pre-specified secondary acute utilization outcomes included any overnight hospital stay over the same 12-month reference period, while recurrent ED visits (defined as two or more visits) were analyzed as a pre-specified sensitivity outcome characterizing high-frequency acute utilization (17). Secondary healthcare access outcomes comprised any reported unmet healthcare need in the past 12 months and forgone mental health care when such services were needed.

Covariates were structured into two pre-specified adjustment specifications, reported side by side rather than treating either as a strict causal estimate. The minimally adjusted model adjusted for baseline sociodemographic characteristics: survey cycle year, child age in continuous years, child sex, race and ethnicity, and household income relative to the federal poverty level. The fully adjusted model additionally incorporated health-need and clinical complexity indicators, including health insurance status, current anxiety, current depression, and a cumulative count of functional difficulties (18, 19). Anxiety, depression, functional difficulties, and insurance status may represent confounders, indicators of clinical severity, or variables on pathways related to healthcare use. Because their role cannot be resolved with cross-sectional data, both specifications are presented to allow readers to assess the association under different adjustment assumptions.

Insurance status was coded in three categories: adequate insurance, inadequate insurance, and uninsured. Uninsured status (NSCH code 95) was retained as a valid analytic category; only item nonresponse (code 99) was treated as missing. In the analytic cohort, 230 children were uninsured (55 in 2022, 72 in 2023, and 103 in 2024), and 25 children had true insurance item nonresponse (5, 9, and 11, respectively).

In addition to insurance status, CSHCN status was defined using the validated CSHCN Screener embedded in the National Survey of Children's Health (NSCH), which operationalizes the federal Maternal and Child Health Bureau definition of CSHCN. A child was classified as CSHCN if the caregiver endorsed at least one of five consequence-based criteria due to a health condition that had lasted or was expected to last ≥12 months: (1) need or use of prescription medication(s); (2) above-routine need or use of medical, mental health, or educational services; (3) functional limitation(s); (4) need or use of specialized therapies; or (5) need for treatment or counseling for an emotional, developmental, or behavioral problem. The exact screener items, response options, and coding are provided in Supplementary Table S8. CSHCN status was used for descriptive characterization and prespecified subgroup analyses; stratified estimates were interpreted as descriptive consistency analyses rather than formal tests of effect modification.

### 2.4 Statistical analysis

All statistical estimations accounted for the complex survey design of the NSCH by incorporating the pooled child weights (single-year sampling weights divided by three), stratification, and household clustering nested within survey cycles. Baseline demographic and clinical characteristics were summarized as survey-weighted percentages or weighted means with corresponding 95% confidence intervals (CIs), stratified by care coordination status (20). Following established methodological guidance for complex observational survey data, descriptive baseline comparisons were reported without comparative inferential *P* values.

Survey-weighted multivariable logistic regression models were fitted to estimate adjusted odds ratios (aORs) and 95% CIs for the association between receipt of needed care coordination and study outcomes (21). For the primary outcome (any ED visit), two pre-specified specifications were reported side by side: the minimally adjusted model (Model 1), which adjusted for survey year, child age, sex, race/ethnicity, and family poverty level; and the fully adjusted model (Model 2), which additionally adjusted for insurance status (adequate, inadequate, or uninsured), current anxiety, current depression, and functional difficulty count. Neither specification was interpreted as a strict causal estimate. Secondary acute utilization outcomes (any overnight hospital stay) and healthcare access outcomes (any unmet healthcare need, forgone mental health care) were evaluated using corresponding fully adjusted survey-weighted models, with recurrent ED use analyzed as a pre-specified sensitivity outcome. To address the risk of type I error across multiple secondary endpoints, *P* values for the family of three secondary outcomes (hospital stay, any unmet healthcare need, and forgone needed mental-health care) were corrected using the Benjamini–Hochberg false discovery rate (FDR) procedure; recurrent ED use was not included in the FDR family (22).

We used model-specific complete-case analyses (23). The minimally adjusted ER model excluded 16 participants (0.3%) with a missing ER outcome, yielding an analytic sample of 6,310. The fully adjusted ER model excluded 181 participants (2.9%) because of a missing outcome or one or more added clinical or access covariates, yielding an analytic sample of 6,145; complete-case sample sizes for secondary and sensitivity outcome models varied slightly (*n* = 6,142–6,147) owing to outcome-specific missingness. Valid uninsured status was retained as an insurance category rather than classified as missing. Because complete-case exclusion may introduce bias if missingness was related to care coordination receipt or healthcare use, this limitation was considered when interpreting results (24).

Pre-specified sensitivity and subgroup analyses evaluated consistency across sub-populations and survey administrations. Year-specific minimally adjusted logistic regression models were estimated separately for 2022, 2023, and 2024, and a formal care-coordination-by-year interaction test was conducted in the pooled sample. Stratified analyses were conducted by Children with Special Health Care Needs (CSHCN) status to assess whether observed associations persisted across both CSHCN and non-CSHCN subgroups; these stratified estimates were descriptive consistency analyses and were not interpreted as formal evidence of effect modification (25). All statistical tests were two-sided, and significance was determined at an alpha threshold of 0.05. Taylor series linearization was employed across all model specifications to calculate appropriate design-corrected standard errors.

## 3 Results

### 3.1 Study cohort and population clinical profile

The analytic cohort comprised an unweighted sample of 6,326 children aged 6 to 17 years with caregiver-reported chronic physical pain and verified care coordination needs, representing a weighted national estimate of approximately 878,491 children per survey year (Supplementary Table S1). The annual contributions to the pooled cohort ranged from 1,930 (2023) to 2,402 (2024) unweighted respondents. Table 1 summarizes the baseline characteristics of the study population by care coordination status. Caregivers who received needed coordination reported a clinical profile that contrasted with those facing unmet coordination needs. While income poverty distributions were comparable across groups, with approximately 21% of both groups residing below the federal poverty line, children with unmet coordination needs carried a higher baseline burden of systemic and clinical vulnerabilities. Specifically, these children exhibited lower insurance adequacy (48.1% vs. 68.5%), higher prevalence of current anxiety (49.9% vs. 35.9%) and depression (26.6% vs. 20.3%), greater functional difficulties (mean 2.7 vs. 2.1), and higher qualification for Children with Special Health Care Needs status (70.0% vs. 55.4%). These baseline differences indicate that children with unmet coordination needs carried a higher observed clinical burden, which motivated the fully adjusted specification described in the Methods and underscores the importance of interpreting both models together.

**Table 1:** Baseline characteristics of the study population by receipt of care coordination.

Characteristic	Did not receive needed coordination(weighted % or mean [95% CI])	Received needed coordination(weighted % or mean [95% CI])
Demographics		
Age, years, mean (95% CI)	12.8 (12.5–13.0)	13.3 (13.0–13.5)
Sex, weighted % (95% CI)		
Male	38.1 (34.8–41.4)	40.0 (36.5–43.4)
Female	61.9 (58.6–65.2)	60.0 (56.6–63.5)
Race and ethnicity, weighted % (95% CI)		
White	74.3 (71.2–77.3)	69.5 (65.8–73.1)
Black	13.3 (10.6–15.9)	18.8 (15.3–22.2)
Other race/multiracial	12.5 (10.6–14.3)	11.8 (9.9–13.6)
Socioeconomic status		
Family poverty level, weighted % (95% CI)		
0%–99% FPL	21.6 (18.3–25.0)	21.1 (17.6–24.7)
100%–199% FPL	23.2 (20.1–26.4)	23.4 (19.8–27.0)
200%–399% FPL	29.5 (26.2–32.8)	27.7 (24.5–31.0)
≥400% FPL	25.7 (22.9–28.4)	27.7 (24.7–30.7)
Healthcare & clinical profile		
Insurance adequacy, weighted % (95% CI)		
Not adequate	51.9 (48.2–55.7)	31.5 (27.8–35.2)
Adequate	48.1 (44.3–51.8)	68.5 (64.8–72.2)
Current anxiety, weighted % (95% CI)		
No current anxiety	50.1 (46.4–53.8)	64.1 (60.5–67.8)
Current anxiety	49.9 (46.2–53.6)	35.9 (32.2–39.5)
Current depression, weighted % (95% CI)		
No current depression	73.4 (70.4–76.4)	79.7 (75.9–83.5)
Current depression	26.6 (23.6–29.6)	20.3 (16.5–24.1)
Special health care needs, weighted % (95% CI)		
Non-CSHCN	30.0 (26.3–33.8)	44.6 (40.8–48.5)
CSHCN	70.0 (66.2–73.7)	55.4 (51.5–59.2)
Functional difficulty count, mean (95% CI)	2.7 (2.6–2.8)	2.1 (1.9–2.3)

Data are presented as survey-weighted % (95% CI) or mean (95% CI). Percentages may not sum to 100% due to rounding. *P* values are not presented because these characteristics are descriptive comparisons of exposure-defined groups; absolute standardized differences may be provided to characterize imbalance.

CI, confidence interval; CSHCN, children with special health care needs; FPL, federal poverty level.

### 3.2 Weighted healthcare utilization and access outcomes by care coordination status

Receipt of needed care coordination was accompanied by consistently lower weighted prevalence of acute healthcare encounters and health-system access barriers (Figure 2, Supplementary Table S6). The weighted prevalence of any ED use differed by 11.0 percentage points between children who did not receive needed coordination (40.0%; 95% CI 36.4–43.7) and those who did (29.0%; 95% CI 25.4–32.5). Recurrent emergency room visits of two or more encounters were reported by 17.5% (95% CI 14.7–20.3) versus 10.3% (95% CI 7.3–13.2), and inpatient hospital stays by 11.4% (95% CI 8.5–14.2) versus 7.1% (95% CI 4.3–9.9). Access deficits were also less frequently reported among children who received coordination: any unmet healthcare need was reported by 20.5% (95% CI 17.9–23.1) versus 6.6% (95% CI 4.9–8.3), and forgone needed mental health care by 12.6% (95% CI 10.4–14.8) versus 2.1% (95% CI 1.2–3.0).

**Figure 2:**
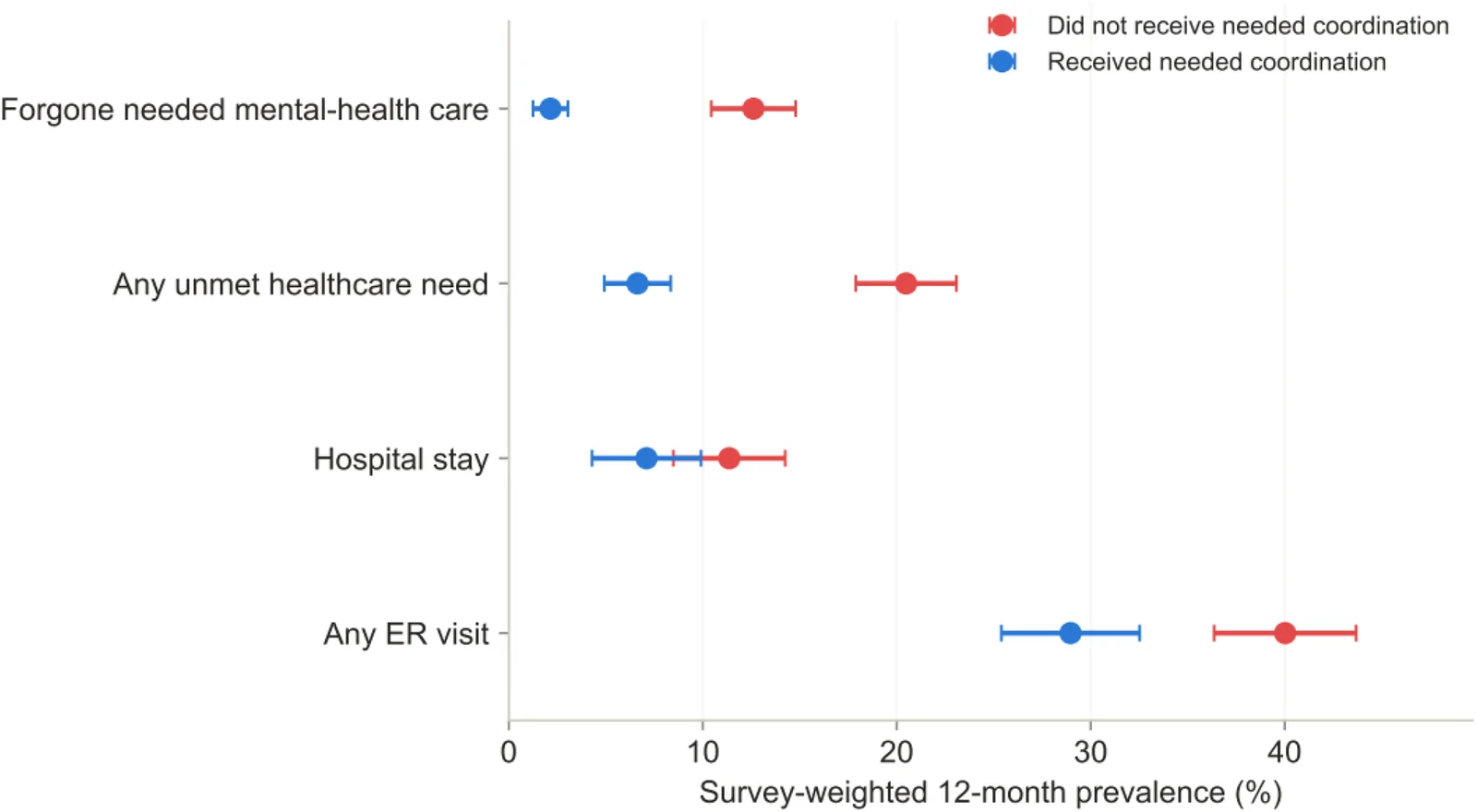
Healthcare utilization and access outcomes by receipt of needed care coordination among United States children with chronic physical pain, 2022–2024.

### 3.3 Minimally adjusted and fully adjusted models for acute care utilization

In multivariable survey-weighted logistic regression, receipt of needed care coordination was associated with lower odds of any ER use in the minimally adjusted model (aOR 0.61; 95% CI 0.48–0.76; *P* < .001; *n* = 6,310). The inverse association persisted after additional adjustment for insurance status, anxiety, depression, and functional difficulties in the fully adjusted model (aOR 0.69; 95% CI 0.54–0.87; *P* = .0019; *n* = 6,145). When assessing high-frequency acute utilization as a pre-specified sensitivity outcome, care coordination was associated with lower odds of recurrent emergency visits (aOR 0.54; 95% CI 0.36–0.79; *P* = .0015; *n* = 6,145). The estimate for inpatient hospitalization was imprecise and included the null (aOR 0.61; 95% CI 0.37–1.01; *P* = .053; *n* = 6,143). Complete parameter estimates for both specifications are provided in Supplementary Table S4. Model results are summarized in Table 2 and Figure 3.

**Table 2:** Adjusted associations of care coordination With healthcare outcomes.

Outcome and model specification	Adjusted OR (95% CI)	*P* value	FDR-adjusted *P* value (q value)
Primary outcome			
Any ER use (minimally adjusted model; *n* = 6,310)	0.61 (0.48–0.76)	<.001	—
Any ER use (fully adjusted model; *n* = 6,145)	0.69 (0.54–0.87)	0.0019	—
Secondary and sensitivity outcomes			
Recurrent ER use (≥2 visits; fully adjusted model; *n* = 6,145)	0.54 (0.36–0.79)	0.0015	—
Hospital stay (fully adjusted model; imprecise estimate; *n* = 6,143)	0.61 (0.37–1.01)	0.053	0.053
Any unmet healthcare need (fully adjusted model; *n* = 6,147)	0.32 (0.23–0.45)	<.001	<.001
Forgone needed mental-health care (fully adjusted model; *n* = 6,142)	0.21 (0.13–0.33)	<.001	<.001

The reference group for all models was children who needed care coordination but did not receive it. The minimally adjusted model included survey year, child age, sex, race/ethnicity, and family poverty level. Fully adjusted models additionally included insurance status (adequate, inadequate, or uninsured), current anxiety, current depression, and functional difficulty count. Recurrent ER use was analyzed as a pre-specified sensitivity outcome and was not included in the false discovery rate (FDR) correction. FDR adjustment using the Benjamini–Hochberg procedure was applied to hospital stay, any unmet healthcare need, and forgone needed mental-health care.

aOR, adjusted odds ratio; CI, confidence interval; ER, emergency room; FDR, false discovery rate.

**Figure 3:**
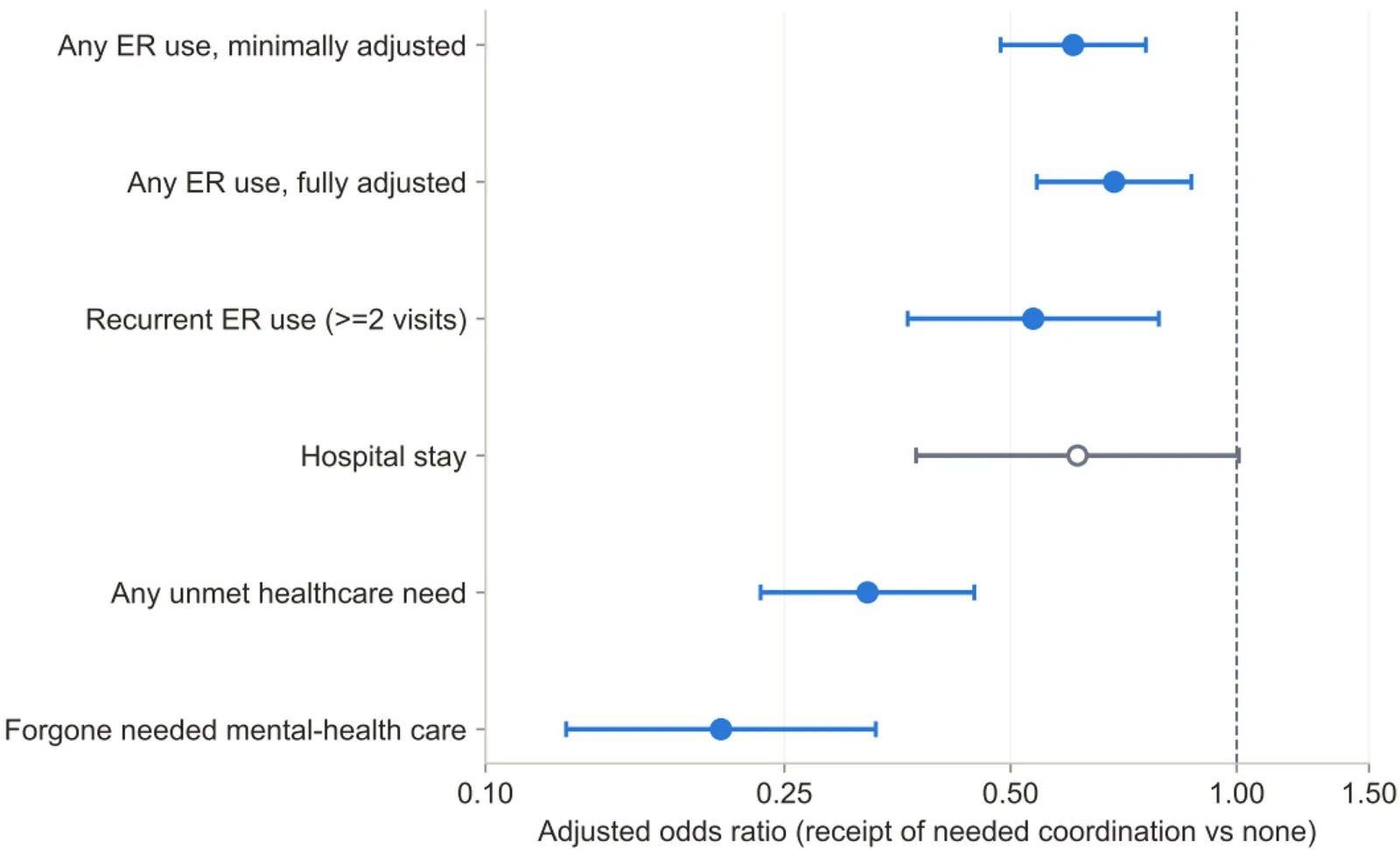
Association of receipt of care coordination with healthcare utilization and access outcomes among US children with chronic pain.

### 3.4 Healthcare access outcomes

Beyond acute hospital-based utilization, receipt of needed care coordination was associated with lower reported odds of unmet healthcare needs and forgone mental-health care. Under the Benjamini–Hochberg false discovery rate procedure, care coordination was associated with a 68% reduction in the odds of any unmet healthcare need (aOR 0.32; 95% CI 0.23–0.45; q < .001) and a 79% reduction in the odds of forgoing necessary mental health care (aOR 0.21; 95% CI 0.13–0.33; q < .001). Because these are related care-access measures assessed during the same recall period, the findings should not be interpreted as independent causal outcomes; temporal ordering cannot be established from these cross-sectional data.

### 3.5 Subgroup generalizability and multi-year stability

Pre-specified subgroup and sensitivity evaluations examined consistency across clinical profiles and survey waves. Stratification by special healthcare needs status yielded inverse associations in both children classified as CSHCN (aOR 0.76; 95% CI 0.59–0.98) and children without this designation (non-CSHCN: aOR 0.48; 95% CI 0.32–0.72) (Supplementary Table S3 and Supplementary Figure S2). These stratified estimates are descriptive consistency analyses and were not interpreted as formal evidence of effect modification.

In year-specific minimally adjusted models, point estimates were below 1.0 in each survey year; the association reached conventional statistical significance in 2023 (aOR 0.54; 95% CI 0.37–0.78; *P* = .001; *n* = 1,922) and 2024 (aOR 0.49; 95% CI 0.35–0.69; *P* < .001; *n* = 2,397) but not in 2022 (aOR 0.78; 95% CI 0.53–1.15; *P* = .204; *n* = 1,991). The care-coordination-by-year interaction was not statistically significant (*P* = .176), providing no clear statistical evidence that the association differed across survey years; these analyses should therefore be interpreted as year-specific estimates rather than evidence of progressive strengthening over time (Supplementary Table S2 and Supplementary Figure S1).

## 4 Discussion

In this nationally representative study of US children with chronic physical pain who required care coordination, receiving needed coordination was consistently associated with substantially lower odds of all-cause emergency department utilization. This inverse association was observed in both the minimally adjusted specification and the fully adjusted model that accounted for insurance status, psychiatric comorbidities, and functional limitations. The association was most pronounced for recurrent emergency visits, where receiving needed coordination was associated with an approximate 46% reduction in odds. Taken together, these findings suggest that acute hospital-based encounters in pediatric chronic pain do not merely reflect disease severity, but may also signal addressable vulnerabilities in outpatient healthcare delivery.

The observed associations between care coordination and secondary access outcomes are consistent with potential pathways linking fragmented care to acute service use. Chronic physical pain in youth often requires simultaneous input from pediatric primary care, subspecialty medicine, physical therapy, behavioral health, and educational systems (2). When structured coordination is absent, families often face significant administrative friction, which may contribute to delayed interventions and crisis-driven visits to the emergency department. In our cohort, children who received needed coordination had lower reported odds of experiencing unmet general healthcare needs and forgone mental health care. In contrast, the estimate for inpatient hospitalization was imprecise and included the null. This discrepancy is clinically coherent: emergency department visits frequently function as an ambulatory safety net for unmanaged pain flares, medication questions, and family distress, whereas inpatient admissions are primarily governed by acute medical instability, complex diagnostic workups, or planned surgical procedures that may be less sensitive to routine outpatient coordination.

Subgroup and sensitivity analyses indicate that the observed associations extend across different clinical profiles (26). Stratification by special healthcare needs status showed inverse associations both among children qualifying as children with special healthcare needs (CSHCN) and among those without this formal designation, with a stronger point estimate among non-CSHCN youth. This pattern suggests that chronic pain alone may introduce substantial management complexity: even in the absence of generalized multi-organ medical complexity, the burden of persistent pain may overwhelm routine care pathways. These stratified estimates are descriptive and should not be interpreted as formal evidence of effect modification.

From a health services perspective, these findings suggest that care coordination may be an important component of pediatric pain management (27, 28). Pediatric chronic pain programs that incorporate designated care managers, proactive communication protocols between specialists and primary care clinicians, and expedited behavioral health referrals may alleviate operational strain on emergency departments. If replicated in longitudinal or interventional studies, these findings may inform the design and evaluation of integrated pediatric pain-care pathways and systemic incentives such as value-based pediatric alternative payment models and dedicated reimbursement codes for non-face-to-face care management, which currently remain underutilized or insufficiently compensated in many state Medicaid and commercial insurance programs (28).

This study has several important limitations that must be considered when interpreting the results. First, the National Survey of Children's Health relies on a cross-sectional design, which precludes establishing causal relationships or temporal ordering. Because both care coordination receipt and emergency department use share a 12-month recall window, we cannot ascertain whether coordination preceded or followed acute encounters (29, 30). Second, we relied on model-specific complete-case analyses; complete-case exclusion may introduce bias if missingness was related to care coordination receipt or healthcare utilization (31, 32). Although exclusions were small (0.3% in the minimally adjusted model and 2.9% in the fully adjusted model), this assumption cannot be formally verified. Third, all measures were based on caregiver report rather than medical records or administrative claims, introducing the potential for recall bias and misclassification. Fourth, emergency department use was measured as all-cause utilization rather than pain-specific encounters, meaning some visits were prompted by acute injuries or unrelated illnesses. Fifth, the survey lacks clinical granularity regarding pain duration, anatomical distribution, numerical severity scores, underlying medical etiologies, and specific therapeutic regimens. Additionally, approximately 92% of children with a valid chronic-pain response were excluded because their caregivers did not report chronic physical pain. Supplementary Table S5 shows that these children differed substantially from children with caregiver-reported chronic pain: they were younger, less likely to be female, less likely to have current anxiety or depression, less likely to be CSHCN, and had fewer functional difficulties and lower healthcare utilization. This selection may limit generalizability to children with milder, unrecognized, or unreported pain. However, because the primary association was estimated within children who reported chronic pain and needed care coordination, this selection is unlikely to fully explain the observed inverse association, although residual selection bias cannot be excluded. Finally, our study was restricted to children whose caregivers explicitly identified a need for care coordination; consequently, these findings may not generalize to children with milder symptoms or those whose families navigate care without perceived need for formal coordination support (33).

## 5 Conclusion

Among US children with chronic physical pain who needed care coordination, receipt of needed care coordination was associated with lower odds of overall emergency department use, recurrent emergency visits, and unmet medical and behavioral healthcare needs in both minimally adjusted and fully adjusted models. These findings underscore the potential clinical importance of addressing fragmented service delivery in pediatric chronic pain care. If replicated in longitudinal or interventional studies, they may inform the design and evaluation of integrated pediatric pain-care pathways, including dedicated care coordination infrastructure, multidisciplinary communication networks, and aligned payment models.

## Data Availability

Publicly available datasets were analyzed in this study. This data can be found here: https://www.childhealthdata.org.

## References

[1] Chambers CT Dol J Tutelman PR Langley CL Parker JA Cormier BT. The prevalence of chronic pain in children and adolescents: a systematic review update and meta-analysis. Pain. (2024) 165(10):2215–34. 10.1097/j.pain.0000000000003267 38743558 PMC11404345

[2] Sommers E D'Amico S Goldstein L Gardiner P. Integrative approaches to pediatric chronic pain in an urban safety-net hospital: cost savings, clinical benefits, and safety. J Integr Complement Med. (2022) 28(5):445–53. 10.1089/jicm.2021.0296 35285677

[3] Stoopler M Choinière M Nam A Guigui A Walfish L Mohamed N. Chronic pain-related consultations to the emergency department of children with complex pain conditions: a retrospective analysis of healthcare utilization and costs. Can J Pain. (2022) 6(1):86–94. 10.1080/24740527.2022.2070840 35756890 PMC9225287

[4] Mahrer NE Gold JI Luu M Herman PM. A cost-analysis of an interdisciplinary pediatric chronic pain clinic. J Pain. (2018) 19(2):158–65. 10.1016/j.jpain.2017.09.008 29054492

[5] Gerhold K Al-Azazi S El-Matary W Katz LY Lim LSH Marks SD. Health care utilization and direct costs prior to subspecialty care in children with chronic pain compared with other chronic childhood diseases: a cohort study. J Pediatr. (2024) 271:114046. 10.1016/j.jpeds.2024.114046 38582149

[6] Cohen E Kuo DZ Agrawal R Berry JG Bhagat SK Simon TD. Children with medical complexity: an emerging population for clinical and research initiatives. Pediatrics. (2011) 127(3):529–38. 10.1542/peds.2010-0910 21339266 PMC3387912

[7] Pulcini CD Coller RJ Houtrow AJ Belardo Z Zorc JJ. Preventing emergency department visits for children with medical complexity through ambulatory care: a systematic review. Acad Pediatr. (2021) 21(4):605–16. 10.1016/j.acap.2021.01.006 33486099

[8] Coller RJ Nelson BB Sklansky DJ Saenz AA Klitzner TS Lerner CF. Preventing hospitalizations in children with medical complexity: a systematic review. Pediatrics. (2014) 134(6):e1628–1647. 10.1542/peds.2014-1956 25384492

[9] Groenewald CB Essner BS Wright D Fesinmeyer MD Palermo TM. The economic costs of chronic pain among a cohort of treatment-seeking adolescents in the United States. J Pain. (2014) 15(9):925–33. 10.1016/j.jpain.2014.06.002 24953887 PMC4150826

[10] Tumin D Drees D Miller R Wrona S Hayes D Tobias JD. Health care utilization and costs associated with pediatric chronic pain. J Pain. (2018) 19(9):973–82. 10.1016/j.jpain.2018.03.012 29608973

[11] Kapos FP Vandeleur DM Tham SW Palermo TM Groenewald CB. Comparing the prevalence of chronic pain in school-aged children in the United States from 2019 to 2020: a nationally representative study examining differences associated with the COVID-19 pandemic. Pain. (2024) 165(1):233–42. 10.1097/j.pain.0000000000003020 37556380 PMC10841312

[12] Casseus M Reichman NE. Trends and patterns in United States pediatric care coordination, 2016-2022. J Pediatr. (2025) 279:114457. 10.1016/j.jpeds.2024.114457 39733831

[13] Vandeleur DM Palermo TM. Transition of care readiness among adolescents with chronic pain between 2021 and 2022 in a nationally representative sample. J Pain. (2025) 29:105333. 10.1016/j.jpain.2025.105333 39929354 PMC11925652

[14] Van Dyck P Kogan MD Heppel D Blumberg SJ Cynamon ML Newacheck PW. The national survey of children’s health: a new data resource. Matern Child Health J. (2004) 8(3):183–8. 10.1023/B:MACI.0000037693.09847.f6 15499874

[15] Ghandour RM Jones JR Lebrun-Harris LA Minnaert J Blumberg SJ Fields J. The design and implementation of the 2016 National Survey of Children’s Health. Matern Child Health J. (2018) 22(8):1093–102. 10.1007/s10995-018-2526-x 29744710 PMC6372340

[16] Toomey SL Chien AT Elliott MN Ratner J Schuster MA. Disparities in unmet need for care coordination: the national survey of children’s health. Pediatrics. (2013) 131(2):217–24. 10.1542/peds.2012-1535 23339228

[17] Arthur NSM Blewett LA. Contributions of key components of a medical home on child health outcomes. Matern Child Health J. (2023) 27(3):476–86. 10.1007/s10995-022-03539-7 36460883

[18] Elmore AL Crouch E. The association of adverse childhood experiences with anxiety and depression for children and youth, 8 to 17 years of age. Acad Pediatr. (2020) 20(5):600–8. 10.1016/j.acap.2020.02.012 32092442 PMC7340577

[19] Lollar DJ Hartzell MS Evans MA. Functional difficulties and health conditions among children with special health needs. Pediatrics. (2012) 129(3):e714–722. 10.1542/peds.2011-0780 22371461

[20] Von Elm E Altman DG Egger M Pocock SJ Gøtzsche PC Vandenbroucke JP. The strengthening the reporting of observational studies in epidemiology (STROBE) statement: guidelines for reporting observational studies. J Clin Epidemiol. (2008) 61(4):344–9. 10.1016/j.jclinepi.2007.11.008 18313558

[21] Dey D Haque MS Islam MM Aishi UI Shammy SS Mayen MSA. The proper application of logistic regression model in complex survey data: a systematic review. BMC Med Res Methodol. (2025) 25(1):10. 10.1186/s12874-024-02454-5 39844030 PMC11752662

[22] Benjamini Y Hochberg Y. Controlling the false discovery rate: a practical and powerful approach to multiple testing. J R Stat Soc Ser B Methodol. (1995) 57(1):289–300. 10.1111/j.2517-6161.1995.tb02031.x

[23] Eekhout I De Boer RM Twisk JWR De Vet HCW Heymans MW. Missing data: a systematic review of how they are reported and handled. Epidemiology. (2012) 23(5):729–32. 10.1097/EDE.0b013e3182576cdb 22584299

[24] Groenwold RHH White IR Donders ART Carpenter JR Altman DG Moons KGM. Missing covariate data in clinical research: when and when not to use the missing-indicator method for analysis. CMAJ. (2012) 184(11):1265–9. 10.1503/cmaj.110977 22371511 PMC3414599

[25] Wang R Lagakos SW Ware JH Hunter DJ Drazen JM. Statistics in medicine — reporting of subgroup analyses in clinical trials. N Engl J Med. (2007) 357:2189–94. 10.1056/NEJMsr077003 18032770

[26] Peter Rothwell. Subgroup analysis in randomised controlled trials: importance, indications, and interpretation. Lancet. (2005) 365(9454):176–86. 10.1016/S0140-6736(05)17709-5 15639301

[27] Suleman MI Essuman AA Dada M Williams K. The Johns Hopkins multidisciplinary pediatric pain management service: a concept paper. 10.1080/17581869.2025.2532356 PMC12691566 40653781

[28] Maguire R O'Keeffe N Murray G Flaherty S Lyden N O’Sullivan C. The development of a national interdisciplinary paediatric pain service for children living with complex chronic pain in Ireland. IJIC. (2025) 25:225. 10.5334/ijic.ICIC24103

[29] Hayase T Mieno MN Mori N Yuza Y Sano H Osone S. Inter-reporter differences in symptom burdens in Japanese children with cancer. Pediatr Int. (2024) 66(1):10. 10.1111/ped.15729 38409898

[30] Nicole MA Alexandra G Guolian K Victoria IO Jessica SF Jason H. Agreement between youth and caregiver report of pain and functioning in pediatric sickle cell disease: pedsQL sickle cell disease module. Pain. (2024) 165(3). 10.1097/j.pain.0000000000003079 PMC10859845 37878652

[31] Cornish RP Bartlett JW Macleod J Tilling K. Complete case logistic regression with a dichotomised continuous outcome: a simulation study. 2021.

[32] Lyles RH Allen AS. Missing data in the 2×2 table: patterns and likelihood-based analysis for cross-sectional studies with supplemental sampling. Stat Med. (2003) 22(4):517–34. 10.1002/sim.1348 12590411

[33] Miller R Tumin D Hayes D Uffman JC Raman VT Tobias JD. Unmet need for care coordination among children with special health care needs. Popul Health Manag. (2018) 22:255–61. 10.1089/pop.2018.0094 30272532

