# Supplementary material for: Receipt of needed care coordination and emergency department utilization among US children with chronic physical pain: a pooled analysis of the 2022–2024 National Survey of Children's Health
Source: Front Pain Res (Lausanne). 2026 Oct 7;7:1994046. doi: 10.3389/fpain.2026.1994046 (PMC13682958; doi:10.3389/fpain.2026.1994046)
Supplement: Supplementary file 1 [file Table1.docx]

**Supplemental Materials**

**Supplementary Table 1. Annual and Pooled Analytic Cohort Summary for US Children Aged 6–17 Years With Chronic Physical Pain Who Needed Care Coordination (NSCH 2022–2024)**.

| **Survey Year** | **Unweighted Sample, No. (%)** | **Annual Weighted Population Estimate, N (%)ᵃ** |
| --- | --- | --- |
| **2022** | 1,994 (31.5) | 865,011 (32.8) |
| **2023** | 1,930 (30.5) | 857,658 (32.5) |
| **2024** | 2,402 (38.0) | 912,804 (34.6) |
| *(Annual average)* | — | *(878,491)* |

Abbreviations: NSCH, National Survey of Children’s Health.

Notes:

ᵃ Weighted population estimates represent the national noninstitutionalized population of US children aged 6–17 years with caregiver-reported chronic physical pain who needed care coordination. Values are rounded to the nearest whole integer.

ᵇ The pooled total weighted population reflects the cumulative 3-year weighted person-population (sum of annual estimates: 2,635,472). Per NSCH analytic guidelines, pooled multi-year analyses divided the final single-year child sampling weights by 3, yielding an average annual national estimate of approximately 878,491 children represented per survey year.

**Supplementary Table 2. Year-Specific Minimally Adjusted Associations Between Receipt of Needed Care Coordination and Any ER Visit (NSCH 2022–2024)**.

| Survey Year | Analytic Sample, No.ᵃ | ER Visits (Events), No. (%) | Adjusted OR (95% CI)ᵇ | P Valueᶜ |
| --- | --- | --- | --- | --- |
| 2022 | 1,991 | 606 (30.4) | 0.78 (0.53–1.15) | .204 |
| 2023 | 1,922 | 618 (32.2) | 0.54 (0.37–0.78) | .001 |
| 2024 | 2,397 | 766 (32.0) | 0.49 (0.35–0.69) | <.001 |
| Pooled Minimally Adjusted Model (Overall) | 6,310 | 1,990 (31.5) | 0.61 (0.48–0.76) | <.001 |

Abbreviations: aOR, adjusted odds ratio; CI, confidence interval; ER, emergency room; NSCH, National Survey of Children’s Health.

Notes:

ᵃ Sample size reflects complete cases for the minimally adjusted model (survey-weighted logistic regression adjusting for child age, sex, race/ethnicity, and family poverty level). Sixteen children (0.3% of the pooled cohort of 6,326) were excluded due to a missing ER outcome.

ᵇ The reference category for all models is children who needed but did not receive care coordination (no coordination = reference [1.00]). All estimates incorporate single-year child sampling weights, stratification, and household clustering.

ᶜ Two-sided P values derived from survey-weighted Wald tests. A formal test for the care-coordination-by-year interaction in the pooled sample was not statistically significant (P for interaction = .176), providing no clear statistical evidence that the association differed across survey years.

**Supplementary Table 3. Minimally Adjusted Multivariable Associations Between Receipt of Needed Care Coordination and Any Emergency Room Visit, Stratified by Special Health Care Needs Status (NSCH 2022–2024)**.

| **Subgroup (CSHCN Status)** | **Unweighted Cohort, No. (%)ᵃ** | **ER Visits (Events), No. (%)** | **Adjusted Odds Ratio (95% CI)ᵇ** | ***P* Valueᶜ** |
| --- | --- | --- | --- | --- |
| **CSHCN** | 4,417 (70.0) | 1,554 (35.2) | 0.76 (0.59–0.98) | 0.03 |
| **Non-CSHCN** | 1,893 (30.0) | 436 (23.0) | 0.48 (0.32–0.72) | <.001 |
| *Pooled Minimally Adjusted Model (Overall)* | *6,310 (100.0)* | *1,990 (31.5)* | *0.61 (0.48–0.76)* | *<.001* |

Abbreviations: aOR, adjusted odds ratio; CI, confidence interval; CSHCN, children with special health care needs; ER, emergency room; NSCH, National Survey of Children’s Health.

Notes:

ᵃ Subgroups were classified using the validated CSHCN Screener embedded in the NSCH. The stratified analytic sample totaled 6,310 children; 16 respondents in the overall cohort (N = 6,326) were excluded due to a missing ER outcome in the minimally adjusted model.

ᵇ Reference category for all models is children who needed but did not receive care coordination (no coordination = reference [1.00]). Each stratum-specific model adjusted for child age (continuous), sex (female vs male), race/ethnicity (non-Hispanic White, non-Hispanic Black, and other/multiracial), family poverty level (<100%, 100%–199%, 200%–399%, and ≥400% of the federal poverty level), and survey year (2022, 2023, 2024). All estimates incorporate survey weights, stratification, and household clustering nested within year.

ᶜ Two-sided P values derived from survey-weighted Wald tests. Stratified estimates were descriptive consistency analyses and were not interpreted as formal evidence of effect modification.

**Supplementary Table 4. Full Parameter Estimates from Multivariable Survey-Weighted Logistic Regression Models for Any Emergency Room Visit (NSCH 2022–2024)**.

| **Independent Variable** | **Minimally Adjusted ER Modelᵃ** |  |  | **Fully Adjusted ER Modelᵇ** |  |  |
| --- | --- | --- | --- | --- | --- | --- |
|  | Beta (SE) | aOR (95% CI) | P Value | Beta (SE) | aOR (95% CI) | P Value |
| Intercept | 0.16 (0.28) | 1.18 (0.68–2.05) | 0.561 | −0.26 (0.32) | 0.77 (0.41–1.43) | 0.404 |
| **Care coordination received** |  |  |  |  |  |  |
| No (needed but did not receive) | 0 [Reference] | 1.00 [Reference] | — | 0 [Reference] | 1.00 [Reference] | — |
| Yes (received needed coordination) | −0.50 (0.11) | 0.61 (0.48–0.76) | <.001 | −0.38 (0.12) | 0.69 (0.54–0.87) | 0.0019 |
| **Survey year** |  |  |  |  |  |  |
| 2022 | 0 [Reference] | 1.00 [Reference] | — | 0 [Reference] | 1.00 [Reference] | — |
| 2023 | 0.09 (0.15) | 1.09 (0.81–1.47) | 0.556 | 0.09 (0.15) | 1.09 (0.82–1.46) | 0.555 |
| 2024 | 0.14 (0.14) | 1.15 (0.87–1.53) | 0.321 | 0.13 (0.14) | 1.14 (0.86–1.51) | 0.36 |
| Child age, per 1-year increase | −0.01 (0.02) | 0.99 (0.95–1.02) | 0.5 | −0.02 (0.02) | 0.98 (0.94–1.01) | 0.241 |
| **Child sex** |  |  |  |  |  |  |
| Male | 0 [Reference] | 1.00 [Reference] | — | 0 [Reference] | 1.00 [Reference] | — |
| Female | −0.01 (0.11) | 0.99 (0.80–1.23) | 0.951 | 0.04 (0.11) | 1.04 (0.83–1.30) | 0.742 |
| **Race and ethnicity** |  |  |  |  |  |  |
| Non-Hispanic White | 0 [Reference] | 1.00 [Reference] | — | 0 [Reference] | 1.00 [Reference] | — |
| Non-Hispanic Black | 0.01 (0.18) | 1.01 (0.71–1.43) | 0.958 | 0.00 (0.19) | 1.00 (0.69–1.46) | 0.982 |
| Other/Multiracial | −0.25 (0.14) | 0.78 (0.59–1.02) | 0.073 | −0.25 (0.14) | 0.78 (0.59–1.02) | 0.074 |
| **Family poverty level (% FPL)** |  |  |  |  |  |  |
| 0%–99% FPL | 0 [Reference] | 1.00 [Reference] | — | 0 [Reference] | 1.00 [Reference] | — |
| 100%–199% FPL | −0.05 (0.18) | 0.95 (0.67–1.36) | 0.783 | 0.00 (0.19) | 1.00 (0.69–1.46) | 0.997 |
| 200%–399% FPL | −0.82 (0.17) | 0.44 (0.32–0.61) | <.001 | −0.76 (0.17) | 0.47 (0.33–0.65) | <.001 |
| ≥400% FPL | −0.84 (0.16) | 0.43 (0.31–0.59) | <.001 | −0.75 (0.17) | 0.47 (0.34–0.66) | <.001 |
| **Insurance status** |  |  |  |  |  |  |
| Inadequate insurance | — | — | — | 0 [Reference] | 1.00 [Reference] | — |
| Adequate insurance | — | — | — | −0.17 (0.13) | 0.84 (0.65–1.09) | 0.192 |
| Uninsured | — | — | — | −0.39 (0.31) | 0.68 (0.37–1.25) | 0.213 |
| **Current anxiety** |  |  |  |  |  |  |
| No | — | — | — | 0 [Reference] | 1.00 [Reference] | — |
| Yes | — | — | — | −0.05 (0.16) | 0.95 (0.70–1.29) | 0.737 |
| **Current depression** |  |  |  |  |  |  |
| No | — | — | — | 0 [Reference] | 1.00 [Reference] | — |
| Yes | — | — | — | 0.27 (0.20) | 1.31 (0.89–1.93) | 0.166 |
| Functional difficulties count, per 1-difficulty increase | — | — | — | 0.19 (0.04) | 1.21 (1.11–1.32) | <.001 |

Abbreviations: aOR, adjusted odds ratio; CI, confidence interval; ER, emergency room; FPL, federal poverty level; NSCH, National Survey of Children's Health; SE, standard error.

Notes:

ᵃ The minimally adjusted ER model adjusted for survey year, child age (continuous), child sex, race/ethnicity, and family poverty level. All estimates were derived using survey-weighted logistic regression accounting for complex design features (pooling weights divided by 3, strata, and household clusters nested within survey year). Complete-case sample size: n = 6,310.

ᵇ The fully adjusted ER model additionally incorporated insurance status (adequate, inadequate, or uninsured; valid uninsured status [NSCH code 95] was retained as a distinct category rather than classified as missing), current anxiety, current depression, and total functional difficulty count. Complete-case sample size: n = 6,145. Because these clinical and access variables may represent confounders, severity indicators, or variables on pathways related to healthcare use, both models are reported side by side rather than treating either as a strict causal estimate.

**Supplementary Table 5. Comparison of children with caregiver-reported chronic physical pain versus those without caregiver-reported chronic physical pain among children with a valid chronic-pain response, National Survey of Children’s Health, 2022–2024.**

| **Characteristic** | **Caregiver-reported chronic physical pain (unweighted n = 8,342)** | **No caregiver-reported chronic physical pain (unweighted n = 96,612)** | **Design-based P value** |
| --- | --- | --- | --- |
| **Sociodemographic characteristics** |  |  |  |
| Age, years, mean (95% CI) | 13.1 (12.9–13.2) | 11.5 (11.5–11.5) | < .001 |
| Female, % (95% CI) | 61.2 (59.1–63.3) | 47.9 (47.2–48.5) | < .001 |
| **Race/ethnicity, % (95% CI)** |  |  |  |
| White | 72.0 (70.0–74.0) | 69.6 (69.0–70.2) | 0.021 |
| Black | 14.8 (13.0–16.6) | 13.9 (13.4–14.4) | 0.333 |
| Other/multiracial | 13.2 (11.9–14.4) | 16.5 (16.1–17.0) | < .001 |
| **Family poverty level, % (95% CI)** |  |  |  |
| <100% FPL | 23.2 (20.9–25.4) | 16.9 (16.3–17.4) | < .001 |
| ≥400% FPL | 25.3 (23.6–27.0) | 34.9 (34.3–35.4) | < .001 |
| **Insurance status, % (95% CI)** |  |  |  |
| Adequate insurance | 55.6 (53.3–57.9) | 65.7 (65.1–66.3) | < .001 |
| Inadequate insurance | 36.1 (34.0–38.3) | 26.6 (26.0–27.1) | < .001 |
| Uninsured | 7.8 (6.3–9.3) | 7.2 (6.8–7.6) | 0.436 |
| **Clinical and health-need characteristics** |  |  |  |
| Current anxiety, % (95% CI) | 37.3 (35.2–39.4) | 11.2 (10.8–11.5) | < .001 |
| Current depression, % (95% CI) | 20.0 (18.1–21.9) | 4.1 (3.9–4.3) | < .001 |
| CSHCN, % (95% CI) | 53.9 (51.6–56.2) | 23.2 (22.7–23.7) | < .001 |
| Functional difficulties, mean (95% CI) | 2.2 (2.2–2.3) | 0.3 (0.3–0.3) | < .001 |
| Needed care coordination, % (95% CI) | 73.1 (71.0–75.1) | 52.7 (52.1–53.4) | < .001 |
| **Healthcare utilization and access outcomes** |  |  |  |
| Any emergency room visit, % (95% CI) | 29.7 (27.5–31.8) | 13.9 (13.5–14.4) | < .001 |
| Any overnight hospital stay, % (95% CI) | 7.3 (5.7–8.8) | 2.2 (2.0–2.4) | < .001 |
| Any unmet healthcare need, % (95% CI) | 12.6 (11.2–13.9) | 3.4 (3.1–3.6) | < .001 |
| Forgone needed mental health care, % (95% CI) | 6.6 (5.6–7.6) | 1.5 (1.3–1.6) | < .001 |

Abbreviations: CI, confidence interval; CSHCN, children with special health care needs; ER, emergency room; FPL, federal poverty level; NSCH, National Survey of Children’s Health.

Notes:

Data are presented as survey-weighted mean (95% CI) for continuous variables and survey-weighted percentage (95% CI) for categorical variables. The pain group includes children with caregiver-reported chronic physical pain; the no-pain group includes children with a valid chronic-pain response but no caregiver-reported chronic physical pain. Estimates incorporate pooled NSCH child sampling weights (single-year weights divided by 3), stratification, and household clustering nested within survey year. P values are design-based and are presented for descriptive comparison.

**Supplementary Table 6. Survey-weighted healthcare utilization and access outcomes by receipt of needed care coordination among US children aged 6–17 years with chronic physical pain who needed care coordination, National Survey of Children’s Health, 2022–2024.**

| **Outcome** | **Did Not Receive Needed Coordination(Weighted % [95% CI])** | **Received Needed Coordination(Weighted % [95% CI])** |
| --- | --- | --- |
| **Acute Healthcare Utilization** |  |  |
| Any ER visit (primary outcome) | 40.0 (36.4–43.7) | 29.0 (25.4–32.5) |
| Recurrent ER use (≥2 visits) | 17.5 (14.7–20.3) | 10.3 (7.3–13.2) |
| Hospital stay | 11.4 (8.5–14.2) | 7.1 (4.3–9.9) |
| **Healthcare Access** |  |  |
| Any unmet healthcare need | 20.5 (17.9–23.1) | 6.6 (4.9–8.3) |
| Forgone needed mental-health care | 12.6 (10.4–14.8) | 2.1 (1.2–3.0) |

Abbreviations: CI, confidence interval; ER, emergency room; NSCH, National Survey of Children’s Health.

Notes:

Data are presented as survey-weighted percentages with 95% confidence intervals. All utilization and access outcomes pertain to the preceding 12 months. Survey weights, stratification, and household clustering were incorporated to reflect US national population estimates.

Abbreviations: CI, confidence interval; ER, emergency room.

**Supplementary Table 7. National Survey of Children’s Health items, variable names, response options, and coding used in the present study, 2022–2024.**

| **Construct** | **NSCH survey question** | **Variable nameᵃ** | **Response options** | **Coding in present study** |
| --- | --- | --- | --- | --- |
| **Chronic physical pain** | “During the past 12 months, has this child had frequent or chronic difficulty with any of the following? Repeated or chronic physical pain, including headaches or other back or body pain” | PHYSICALPAIN / DiffPain_1617 | Yes / No | Binary: 1 = caregiver-reported chronic physical pain; 0 = no caregiver-reported chronic physical pain |
| **Care coordination need** | “DURING THE PAST 12 MONTHS, did anyone help you arrange or coordinate this child’s care among the different doctors or services that this child uses?” / “DURING THE PAST 12 MONTHS, did you, another caregiver, or a health care provider need to make any decisions regarding this child’s health care, such as whether to get prescriptions, referrals, or procedures?” | K5Q20 / K5Q20_R / D5 (year-specific) | Yes / No | Caregiver affirmed need for help arranging or coordinating care among ≥2 providers |
| **Care coordination receipt** | Follow-up to care coordination need item: “If yes, how often did parents get as much help as needed arranging or coordinating child’s care” / “Did [child] receive all needed help arranging or coordinating care?” | Year-specific follow-up variable | Yes, all needed / Yes, some / No, not received | Binary: 1 = received all needed care coordination; 0 = needed but did not receive coordination |
| **Any emergency room (ER) visit** | “During the past 12 months, how many times did this child visit a hospital emergency room?” | K4Q01 / ERVisits | 0 times / 1 time / 2 or more times | Binary: 1 = ≥1 ER visit; 0 = no ER visit |
| **Recurrent ER use** | Same item as above | K4Q01 / ERVisits | 0 / 1 / ≥2 times | Binary sensitivity outcome: 1 = ≥2 ER visits; 0 = 0–1 ER visit |
| **Hospital stay** | “During the past 12 months, was this child admitted to the hospital to stay for at least one night?” | HospitalStay_22 / K4Q02 | Yes / No | Binary: 1 = ≥1 overnight hospital stay; 0 = none |
| **Any unmet healthcare need** | “During the past 12 months, was there any time when this child needed health care but it was not received?” | K4Q27 / ForgoneCare_1617 | Yes / No | Binary: 1 = any unmet healthcare need; 0 = none |
| **Forgone mental health care** | “During the past 12 months, was there any time when this child needed mental health care but it was not received?” | K4Q28 / ForgoneMentalHealth / MenthIProb_1617 | Yes / No / Did not need mental health care | Binary among those needing care: 1 = forgone mental health care; 0 = received or did not need |
| **Current anxiety** | “Has a doctor or other health care provider EVER told you that this child has anxiety problems? If yes, does the child currently have the condition, and what would you rate the severity?” | K2Q33A / anxiety_1920 / ANXFREQ_C | Yes, currently / Yes, but not currently / No | Binary: 1 = current anxiety (mild, moderate, or severe); 0 = no current anxiety |
| **Current depression** | “Has a doctor or other health care provider EVER told you that this child has depression? If yes, does the child currently have the condition, and what would you rate the severity?” | K2Q32A / depress_1920 / DEPRESSED | Yes, currently / Yes, but not currently / No | Binary: 1 = current depression (mild, moderate, or severe); 0 = no current depression |
| **Functional difficulties count** | 12 items assessing difficulty with: (1) seeing; (2) hearing; (3) walking/climbing stairs; (4) dressing/bathing; (5) concentrating/remembering/making decisions; (6) communicating; (7) eating/swallowing; (8) using hands/fingers; (9) coordination; (10) chronic pain; (11) breathing; (12) other functional limitation | C_K2Q16 – C_K2Q17 / func_1617 / Diff2more_1617 | Yes / No for each item | Count variable: 0–12 functional difficulties |
| **CSHCN status** | Five CSHCN Screener items: (1) need/use of prescription medication; (2) above-routine need/use of medical, mental health, or educational services; (3) functional limitation; (4) need/use of specialized therapies; (5) need for treatment/counseling for emotional, developmental, or behavioral problem. Each due to condition lasting or expected to last ≥12 months | CSHCN / CSHCNtype_1617 / Screener items K2Q10–K2Q23 | Yes / No for each screener item | Binary: 1 = CSHCN (≥1 screener criterion endorsed); 0 = non-CSHCN |
| **Insurance status** | “Indicate whether any of the following is a reason this child was not covered by health insurance at any time DURING THE PAST 12 MONTHS” / insurance adequacy items | Year-specific insurance variables | Adequate / Inadequate / Uninsured (code 95) / Item nonresponse (code 99) | Categorical: adequate insurance, inadequate insurance, uninsured (code 95 retained as valid category), missing (code 99) |
| **Age** | Derived from household screener and child demographics | SC_AGEYRS | Continuous years | Continuous: child age in years |
| **Sex** | Child sex item | SC_SEX | Male / Female | Binary: female vs male |
| **Race/ethnicity** | NSCH race and Hispanic ethnicity items | SC_RACE_R / SC_HISPANIC_R | Non-Hispanic White / Non-Hispanic Black / Other or multiracial | Categorical: White, Black, Other/multiracial |
| **Family poverty level** | Derived from household income and family size relative to federal poverty level | FPL_I1 / POVERTY_LEVEL | <100% FPL / 100–199% FPL / 200–399% FPL / ≥400% FPL | Categorical: <100%, 100–199%, 200–399%, ≥400% FPL |

Abbreviations: CSHCN, children with special health care needs; ER, emergency room; FPL, federal poverty level; NSCH, National Survey of Children’s Health.

Notes:

ᵃ Variable names may vary across NSCH survey years (2022, 2023, 2024). The most commonly used names are shown; exact names should be verified against the official NSCH codebook for each year.

ᵇ All utilization and access outcomes refer to the 12 months preceding the survey.

ᶜ For anxiety and depression, only children reported to currently have the condition were classified as having the condition; children with a history of the condition but no current diagnosis were classified as not currently having the condition.

ᵈ For the CSHCN Screener, a child was classified as CSHCN if the caregiver endorsed at least one of the five consequences-based criteria due to a health condition that had lasted or was expected to last ≥12 months.

ᵉ Insurance status was coded into three analytic categories: adequate insurance, inadequate insurance, and uninsured. Uninsured status (NSCH code 95) was retained as a valid analytic category; only item nonresponse (code 99) was treated as missing.

ᶠ Item wording may differ slightly across survey years. The wording shown reflects the 2022–2024 production questionnaires. Exact question numbers and skip patterns are available in the official NSCH survey instruments and codebooks at https://www.childhealthdata.org.

**Supplementary Table 8. CSHCN Screener items used to define children with special health care needs in the National Survey of Children’s Health, 2022–2024.**

| **Screener domain** | **Caregiver-reported criterion** | **Response options** | **Coding** | **Duration requirement** |
| --- | --- | --- | --- | --- |
| 1. Prescription medication | Child currently needs or uses medicine prescribed by a doctor, other than vitamins | Yes / No | Yes = 1; No = 0 | Condition has lasted or is expected to last ≥12 months |
| 2. Elevated service use | Child needs or uses more medical care, mental health, or educational services than is usual for most children of the same age | Yes / No | Yes = 1; No = 0 | Condition has lasted or is expected to last ≥12 months |
| 3. Functional limitation | Child is limited or prevented in any way in his or her ability to do the things most children of the same age can do | Yes / No | Yes = 1; No = 0 | Condition has lasted or is expected to last ≥12 months |
| 4. Specialized therapies | Child needs or receives special therapy, such as physical, occupational, or speech therapy | Yes / No | Yes = 1; No = 0 | Condition has lasted or is expected to last ≥12 months |
| 5. Emotional, developmental, or behavioral treatment | Child needs or receives treatment or counseling for an emotional, developmental, or behavioral problem | Yes / No | Yes = 1; No = 0 | Condition has lasted or is expected to last ≥12 months |

Abbreviations: CSHCN, children with special health care needs; NSCH, National Survey of Children’s Health.

Notes:

CSHCN status was defined as a binary indicator. A child was classified as CSHCN if the caregiver endorsed at least one of the five screener domains above and reported that the underlying condition had lasted or was expected to last ≥12 months. The CSHCN Screener is a validated, non-condition-specific instrument that operationalizes the federal Maternal and Child Health Bureau definition of CSHCN. Exact item wording, variable names, and skip patterns should be verified against the official 2022–2024 NSCH questionnaires and codebooks.

**Supplementary Figure 1. Year-specific associations of care coordination with pediatric emergency department use (2022–2024).**

**Supplementary Figure 2. Sensitivity and subgroup analyses of care coordination and emergency department utilization.**
